# A point mutation in the zinc finger motif of RID1/EHD2/OsID1 protein leads to outstanding yield-related traits in *japonica* rice variety Wuyunjing 7

**DOI:** 10.1186/1939-8433-6-24

**Published:** 2013-10-18

**Authors:** Shikai Hu, Guojun Dong, Jie Xu, Yan Su, Zhenyuan Shi, Weijun Ye, Yuanyuan Li, Gengmi Li, Bin Zhang, Jiang Hu, Qian Qian, Dali Zeng, Longbiao Guo

**Affiliations:** State Key Laboratory of Rice Biology, China National Rice Research Institute, Chinese Academy of Agricultural Sciences, Hangzhou, 310006 China

**Keywords:** Heading date, Plant height, Panicle development, Plant architecture, Map-based cloning

## Abstract

**Background:**

Flowering time, which is often associated with the length of the growth period in rice, determines the adaptability of a plant to various environments. However, little is known about how flowering-time genes affect panicle development and yield formation potential in rice after inducing the transition from vegetative growth to reproductive growth.

**Results:**

To explore the relationship between floral induction and yield formation and the molecular mechanism of panicle development in rice, a novel mutant, *ghd10*, was identified from *japonica* variety Wuyunjing 7 plants subjected to ethyl methane sulfonate (EMS) treatment. The *ghd10* mutant exhibited delayed flowering time, tall stalks and increased panicle length and primary branch number. Map-based cloning revealed that *Ghd10* encodes a transcription factor with Cys-2/His-2-type zinc finger motifs. *Ghd10* is orthologous to *INDETERMINATE1 (ID1)*, which promotes flowering in maize (*Zea mays*) and is identical to the previously cloned genes *Rice Indeterminate1 (RID1), Early heading date2 (Ehd2)* and *OsId1*. Transient expression analysis of the Ghd10-GFP fusion protein in tobacco mesophyll cells showed that this protein is expressed in the nucleus. *Ghd10* mRNA accumulated most abundantly in developing leaves and panicle structures, but rarely in roots. Expression analysis revealed that the expression levels of *Ehd1*, *Hd1*, *RFT1*, *Hd3a* and *OsMADS15* decreased dramatically under both short-day and long-day conditions in *ghd10*.

**Conclusion:**

These results indicate that *Ghd10*, which encodes a promoter of flowering, influences plant height and panicle development by regulating the expression levels of some flowering-related genes, such as *Ehd1*, *Hd1*, *OsMADS15* and others. The *ghd10* allele is a useful resource for improvement of panicle traits in rice grown in tropical and low-latitude areas.

**Electronic supplementary material:**

The online version of this article (doi:10.1186/1939-8433-6-24) contains supplementary material, which is available to authorized users.

## Background

Plant architecture is one of the most important agronomic traits affecting rice yield formation potential (Wang and Li 2011). As plant height, heading date and panicle development are important components of plant type and yield potential in rice, the study of these factors will pave the way for clarifying the molecular mechanism of plant morphogenesis and increasing the yield potential of rice using genetic methods (Tripathi et al. 2012; Duan et al. 2013).

Heading date, one of the most important agronomic traits in rice, involves in determining whether currently available varieties of cultivated rice can adapt to specific cropping locations and growing seasons (Izawa 2007; Tsuji et al. 2008). The response to the length of day (LOD) of a rice plant (namely, photoperiod sensitivity [PS]), its temperature sensitivity (TS) and basic vegetative growth (BVG) determine the heading date of the plant. The interaction between these factors is thought to determine adaptability of a rice variety (Lee and An 2007). Many genetic studies of heading date have been performed, and several genes controlling heading date in rice have been mapped or cloned (Andrés et al. 2009; Dai and Xue 2010; Hayama et al. 2003; Matsubara et al. 2008; Park et al. 2008; Saito et al. 2011; Wu et al. 2008; Hu et al. 2012). Yano et al. detected 14 QTLs for heading date (*Hd1-Hd14*) using several types of progeny derived from a single cross of Nipponbare and Kasalath, subsequently leading to the cloning of *Hd1*, *Hd3a* and *Hd6* (Yano et al. 2001). *Hd1*, which is allelic to *Se1*, promotes flowering under short-day (SD) conditions while it represses flowering under long-day (LD) conditions (Yano et al. 2000). *Hd6*, involving in rice photoperiod sensitivity, encodes the α-subunit of protein kinase CK2α (CK2α) to increase the days-to-heading (Takahashi et al. 2001). Another flowering-related gene, *Hd3a* acts as a floral activator under SD conditions and is regulated by *Hd1* (Kojima et al. 2002). The recent studies have shown that Hd3a can interact with 14-3-3 proteins in the apical cells of shoots to yield a complex that is translocated to the nucleus and binds to the OsFD1 transcription factor to induce the transcription of *OsMADS15* (Abe et al. 2005; Taoka et al. 2011). *Ehd1* encodes a B-type response regulator that confers short-day promotion of flowering and controls FT-like gene expression independently of *Hd1* (Doi et al. 2004). Previous studies also found that *Ghd7* and *Ghd8/DTH8* control multiple-traits of flowering time, plant height and grain numbers per panicle simultaneously (Wei et al. 2010; Xue et al. 2008; Yan et al. 2011).

The transition from vegetative growth to reproductive growth marks the successful completion of floral induction. In addition to this transition, the development of panicle morphology is one of the most important processes concerning rice breeders. Many genes influencing panicle morphology and development have been cloned (Huang et al. 2009; Komatsu et al. 2003; Ikeda et al. 2007; Oikawa and Kyozuka 2009; Qiao et al. 2011; Tabuchi et al. 2011; Terao et al. 2010; Yoshida et al. 2012; Zhu et al. 2010). *DEP1* is pleiotropically responsible for dense panicles, high grain number per panicle and erect panicles, and plays an important role in manipulating grain yield (Huang et al. 2009; Yan et al. 2007). Mutation of *SP1* results in plants defective in panicle elongation, thus leads to the short-panicle phenotype (Li et al. 2009). *LAX2 (Lax Panicle2)* is involved in maintaining axillary meristems (AMs) at the reproductive stage. *LAX2* regulates the branching of the aboveground parts of rice throughout plant development, except for the primary branch in the panicle (Komatsu et al. 2001; Komatsu et al. 2003; Oikawa and Kyozuka 2009; Tabuchi et al. 2011). Rice *APO1* encoding an F-box protein was isolated from the *apo1* mutant exhibiting small inflorescence with a reduced number of branches and spikelets, abnormal floral organ identity and a loss of floral determinacy (Ikeda et al. 2007). Habataki-*APO1* rice plants exhibit an increase in the number of primary branches, grain number and yield per plant. APO1 also can enhance the formation of vascular bundle systems, which promotes carbohydrate translocation to panicles and improves lodging resistance in rice (Ookawa et al. 2010). Rice breeders and geneticists have suggested that rice yield formation potential can be increased by further improving rice plant type. *IPA1*, an ideal plant architecture gene, can coordinate the interaction among all of those yield components (Jiao et al. 2010).

Panicle primordium differentiation and stem elongation are important signals for the transition from vegetative growth to reproductive growth. However, there are few reports about how the vegetative phase (before the transition) is connected to panicle morphology development (after the transition), thereby influencing the yield formation potential of rice. In the current study, we investigated the *ghd10* mutant, which exhibits delayed flowering time, tall stalks, increased panicle length and an increased number of primary branches. Map-based cloning revealed that *Ghd10* encodes a transcription factor with Cys-2/His-2-type zinc finger motifs that is orthologous to *INDETERMINATE1 (ID1)*, which promotes flowering in maize (*Zea mays*) and was previously identified as *Rice Indeterminate1 (RID1), Early Heading Date2 (Ehd2)* and *OsId1* in rice. *Ghd10* acts as a promoter of flowering, mainly through the upregulation of *Ehd1* and *Hd1* and the downstream genes *Hd3a*, *RFT1* and others in the unique genetic network of photoperiodic flowering. How *Ghd10* influences plant height, panicle development and even plant architecture has not previously been reported, and the molecular mechanism underlying the activity of *Ghd10* remains unclear. The results of this study suggest that *Ghd10* plays an important role in regulating yield component traits by increasing plant height and primary branch number in rice, which is dependent on short-day conditions.

## Results

### The *ghd10* gain-of-function allele in panicle development

**Figure 1:**
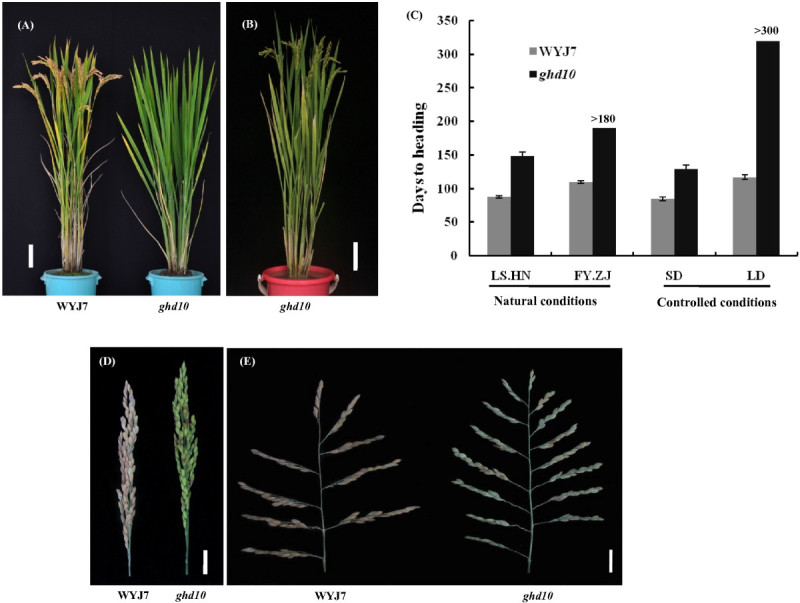
**Phenotype comparison of** ***ghd10*** **mutants and wild-type WYJ7. (A)** The phenotype of wild-type WYJ7 (left) and the *ghd10* mutant (right) at the mature stage (The plants were cultivated at CNRRI experimental field in Fuyang city, Zhejiang Province, in 2011). **(B)** The phenotype of the *ghd10* mutant at the mature stage (The plants were cultivated at CNRRI experimental field in Lingshui city, Hainan Province, in 2011). **(C)** Days to heading comparison between the wild-type and the mutant in Fuyang city, Zhejiang Province (FY.ZJ), Lingshui city, Hainan Province (LS.HN) and under controlled short-day (SD) and long-day (LD) conditions. **(D**–**E)** Increased panicle length **(D)** and primary branch number **(E)** in *ghd10*. Scale bar: **(A)** and **(B)**, 10 cm; **(D)** and **(E)**, 2 cm.

The *ghd10* mutant was obtained from *japonica* cultivar Wuyunjing 7 (WYJ7) rice plants subjected to EMS treatment. Phenotypic analysis showed that the mutant exhibited delayed flowering in both Fuyang, Zhejiang Province (FY.ZJ) and Lingshui, Hainan Province (LS.HN) compared with the wild-type (Figure 1A). The mutant began to flower on the 149th day after germination under natural SD conditions (LS.HN), with an increase of 54 d compared with the wild-type. The mutant never flowered during the more than 300-day period after germination under controlled LD conditions, and it also cannot flower under natural LD conditions (from the middle of May to the middle of November, FY.ZJ), whereas the wild-type plants began to flower on the 110th and 117th day after germination under natural LD conditions (FY.ZJ) and controlled LD conditions, respectively (Figure 1B, C). The difference in flowering time was small in the wild-type plants under SD and LD conditions. Thus, *ghd10* exhibited much later flowering time than the wild type under both SD and LD conditions, and the flowering time delay was more obvious under LD conditions, which indicated that the *Ghd10* mutation may have a more severe effect on flowering time under LD conditions than under SD conditions. And these data were consistent with the finding of previous study (Matsubara et al. 2008; Wu et al. 2008).

**Figure 2:**
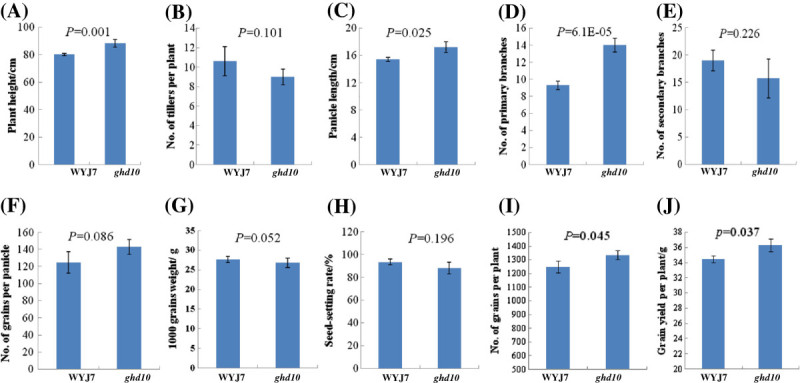
**Agronomic trait performance of** ***ghd10*** **and WYJ7 plants. (A)** Plant height. **(B)** Number of tillers. **(C)** Panicle length. **(D)** Number of primary branch per panicle. **(E)** Number of secondary branch per panicle. **(F)** Number of grains per panicle. **(G)** 1,000-grain weight. **(H)** Seed-setting rate. **(I)** Number of grains per plant. **(J)** Grain yield per plant. WYJ7 and *ghd10* plants were grown in a standard paddy field with a distance of 16*20 cm under conventional cultivation conditions in Lingshui city, Hainan Province, in 2012. All data are shown as mean ± s.e.m. A Student’s t-test was used to generate the P values.

The prolonged vegetative phase in the mutant caused the panicle length and primary branch number of *ghd10* to increase under natural SD conditions compared with the wild type (Figure 1D, E). Detailed agronomic trait analysis revealed that the panicle length of *ghd10* was an average of 17.2 ± 0.8 cm, which was 8.3% longer than that of the wild type (15.4 ± 0.3 cm), there was a significant difference (P < 0.05) between the mutant and wild type. The primary branch number of *ghd10* was 14 ± 0.8, which was higher than that of wild-type WYJ7 (9.3 ± 0.5). The main stem of *ghd10* was approximately 88.3 ± 2.7 cm, which was 10.2% higher than that of the wild type. The primary branch number and plant height were highly significantly different (P < 0.01) between *ghd10* and WYJ7. The number of grains per panicle in the mutant was approximately 143 ± 8.5, which was 12.4% more than that of the wild type (124.7 ± 7.4 on average). These results ran counter to the findings of the previous study (Matsubara et al. 2008; Park et al. 2008; Wu et al. 2008). There was no significant difference in the percentage of seed setting, 1,000-grain weight or tiller number between the *ghd10* mutant and the wild type (Figure 2A–H). These results indicate that the *Ghd10* mutation increases the plant height, panicle length and primary branch number in addition to delaying the heading date.

### Late flowering increases the leaf number per tiller in *ghd10* and is independent of the leaf emergence rate

**Figure 3:**
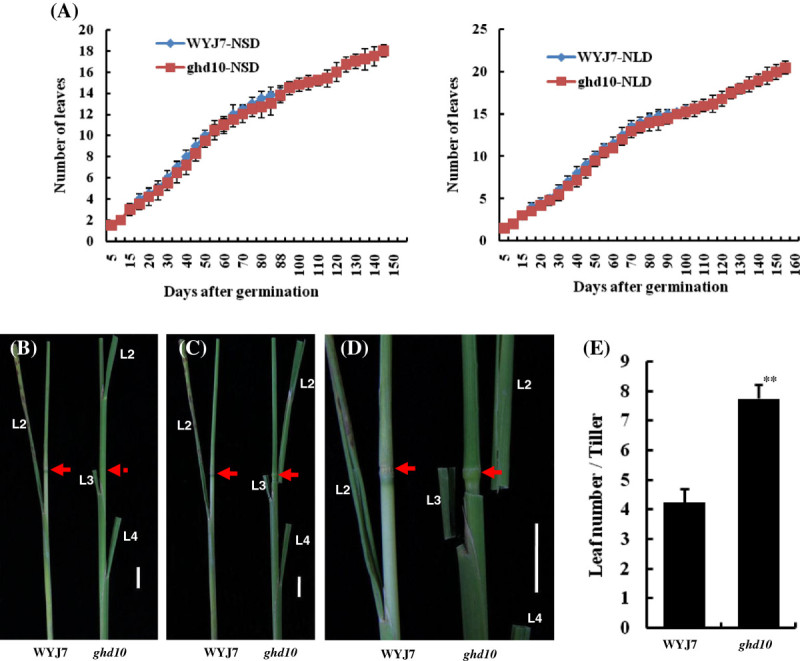
**Comparison of leaf emergence rates between** ***ghd10*** **mutant and wild-type plants. (A)** Comparison of leaf emergence rates. The plants were grown under natural conditions in Fuyang city, Zhejiang Province in 2011. **(B**–**D)** The nodes were wrapped in leaf sheaths in *ghd10* mutant but not in WYJ7. L2, L3 and L4 indicate the leaf number from leaf blade to bottom. Red arrows indicate the uppermost node. Scale bar = 2 cm. **(E)** Leaf number per tiller.

Since the leaf emergence rate reflects the growth and developmental stage or growth rate of rice, we identified the specific growth and developmental periods of the plants by examining the leaf number on the main stem. To determine whether the reduced growth rate affected late flowering in *ghd10*, we compared the leaf emergence rate between *ghd10* and the wild type under natural SD (NSD) and natural LD (NLD) conditions up to the 160th day after germination. Before flowering, the leaf emergence rate of *ghd10* was almost the same as that of the wild type under both NSD and NLD conditions. Under NSD conditions, *ghd10* flowered after the 17th leaf emerged, which occurred by the 149th day after germination, and the mutant developed 21 leaves by the 160th day after germination (Figure 3A). The prolonged heading date increased the number of leaves per tiller in addition to increasing the panicle length and primary branch number (Figure 1C, D), and the nodes were wrapped by the leaf sheath of the additional leaves in *ghd10* under NSD conditions, unlike in WYJ7 (Figure 3B–E). Thus, the mutation of *Ghd10* did not affect the growth rate of the mutant, but the mutation produced a gain-of-function in inflorescence development. These results reveal that *Ghd10* controls the floral transition and the subsequent development of inflorescences in rice.

### The point mutation of *Ghd10* is responsible for the *ghd10* phenotype

To determine whether the *ghd10* phenotype is controlled by a single gene, we performed genetic analysis of reciprocal crosses between *ghd10* and the *japonica* cultivars NIP, WYJ7 and CJ06. The results showed that *ghd10* was a recessive mutant, based on examination of individuals of the F_2_ segregating population. The ratio of individuals with normal flowering to late flowering was 3:1 (χ^2^ < χ^2 0.05,1^ = 3.84), indicating that the *ghd10* phenotype was controlled by a single recessive gene (Additional file 1: Table S2).

**Figure 4:**
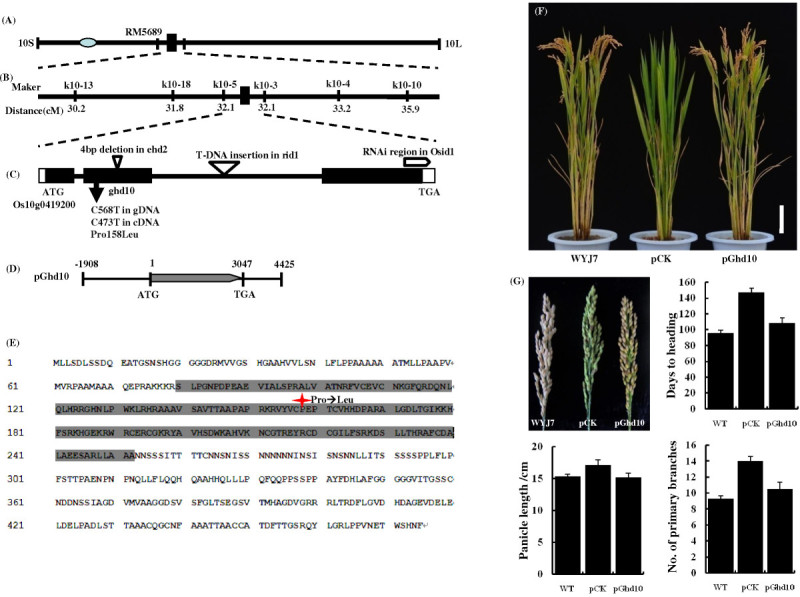
**Map-based cloning of** ***Ghd10*** **. (A)** Location of *Ghd10* on the long arm of rice chromosome 10. **(B)** The gene was mapped to the interval between molecular markers k10-5 and k10-3 on Nipponbare BAC AC027658 on the basis of genotyping 1,647 F_2_ progeny. **(C)** Allelic variation of the *Ghd10* sequence. **(D)** Complementation plasmid. The plasmid pGhd10 contained the entire *Ghd10* gene sequence. **(E)** Gray box indicates the putative ID domain containing two C2H2 and two C2HC zinc fingers. Red asterisk indicates the amino acid substitution in the mutant. **(F)** Phenotypes of transgenic lines. pCK indicates that the empty vector was used in the transgenic line as the negative control. Scale bar = 10 cm. **(G)** Panicle morphology comparison and panicle related-traits analysis among the wild type, negative control and transgenic line. Scale bar =2 cm.

Bulked Segregant Analysis (BSA) was used to produce a primary map of *Ghd10*. PCR genotyping was carried out using a bulk DNA pool from 36 *ghd10*/NJ06 F_2_ individuals with the mutant phenotype, and 136 SSR and STS markers scattered on all of the rice chromosomes were used to determine the approximate map position of *Ghd10*. *Ghd10* was primarily located on chromosome 10, closely linked to RM5689, at a genetic distance of 4.2 cM (Figure 4A). To further fine map *Ghd10*, we designed new STS and InDel markers next to RM5689 based on the sequence difference between *japonica* rice variety Nipponbare and *indica* variety 9311 (http://www.gramene.org/resources/). The polymorphism primers were subsequently used to screen 1,611 individual genotypes, which mapped the *Ghd10* locus between two STS markers, k10-5 and k10-3, within a 29-kb physical interval (Figure 4B). Finally, we obtained one predicted ORF, Os10g0419200, according to the genomic annotation database (RAP-DB, http://rapdb.dna.affrc.go.jp/). Sequence analysis revealed only one single nucleotide polymorphism (SNP) in the Os10g0419200 coding sequence. This mutation resulted in an amino-acid substitution from proline (Pro) to leucine (Leu) in the 158th residue (Figure 4C). Genetic complementation verified the identity of the *Ghd10* candidate gene. We introduced the plasmid pGhd10, which contained the entire Os10g0419200 ORF, and pCK empty vector as a negative control, into *ghd10* (Figure 4D). The phenotype of *ghd10* was restored to normal in the three transgenic pGhd10 lines, whereas all six of the pCK lines of failed to recover the wild type phenotype, which demonstrated that the cloned candidate gene indeed represented *Ghd10* (Figure 4F, G). In summary, we cloned *Ghd10* and determined that a point mutation was responsible for the phenotype of *ghd10*.

### *Ghd10* encodes a putative transcription factor with zinc finger motifs localized in the nucleus

**Figure 5:**
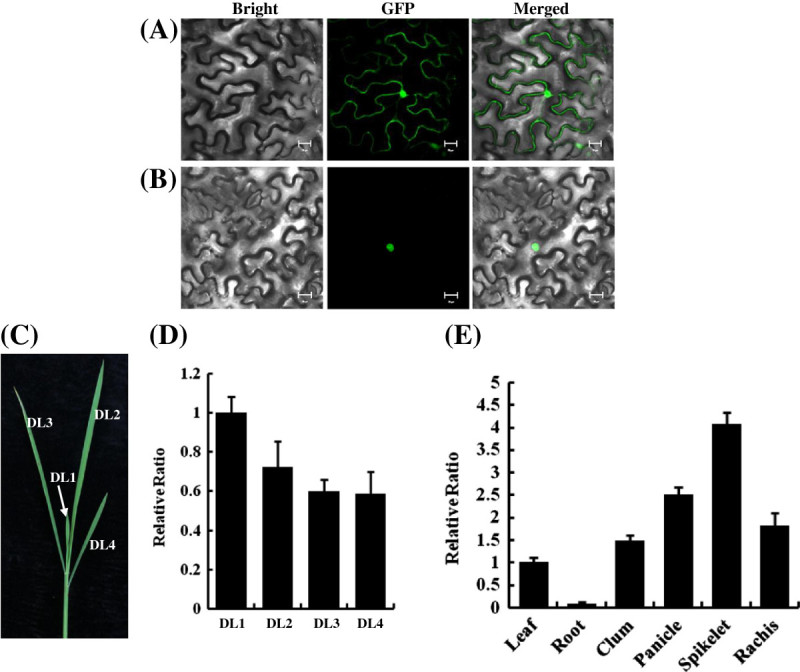
**Expression pattern and subcellular localization. (A**,**B)** Subcellular localization of Ghd10 in tobacco mesophyll cells, **(A)** Empty 35S-GFP vector without a specific targeting sequence, **(B)** GFP signals of the Ghd10-GFP fusion protein. Scale bar = 20 μm. **(C)** Main culms of 5-week-old wild-type plant under natural conditions in Fuyang city, Zhejiang Province, in 2012. **(D)** Expression analysis of *Ghd10* in different developing/developed leaves (DL) using semi-quantitative real time-PCR. **(E)** Quantitative real time-PCR analysis of expression levels of *Ghd10* in roots, culms, leaves, panicles, spikelets and rachises at the heading stage. Error bars (standard deviations) were based on three independent experiments.

Bioinformatic analysis indicated that the coding sequence (CDS) of *Ghd10* consisted of 1,428 nucleotides encoding a 475 amino-acid protein (Figure 4E). The deduced amino acid sequence of Ghd10 contained two C2H2-type and two C2HC-type zinc finger motifs, previously designated as the ID domain (Kozaki et al. 2004), implying that *Ghd10* encoded a zinc finger transcription factor. To assess the subcellular localization of Ghd10, we performed a transient expression experiment to analyze *Ghd10* expression in tobacco epidermal cells. The full-length cDNA was amplified using the following primer pair: CDS-F (5′-ATGTTGCTGTCTGATCTCTCGTCTGA-3′), CDS-R (5′-GAAGTTGTGGCTCCACGTCTCGTTCA-3′). The C terminal of Ghd10 was fused to GFP under the control of the cauliflower mosaic virus (CaMV) 35S promoter, which was injected into the leaves of one-month-old tobacco seedlings. The transformed tobacco plants were incubated for approximately 48 h at 24°C under a 14 h light/10 h dark cycle. Small pieces of injected leaves were then examined under a confocal fluorescence microscope. The results showed that the Ghd10-GFP fusion protein localized exclusively in the nucleus, in contrast to the control, in which GFP signals were observed throughout the tobacco cells (Figure 5A, B). The results of Ghd10-GFP subcellular localization in tobacco mesophyll cells were consistent with the previous study of RID1-GFP in onion epidermal cells (Wu et al. 2008) and OsID1-GFP in roots of transgenic plants (Park et al. 2008).

### Expression pattern of *Ghd10*

Previous studies of the maize *ZmID1* gene revealed that mRNA transcripts of the gene were primarily restricted to young leaves that were folded inside of maize stems (Colasanti et al. 1998; Wong and Colasanti 2007; Matsubara et al. 2008; Park et al. 2008; Wu et al. 2008). Therefore, we first examined the expression levels of *Ghd10* transcripts of young plants at the vegetative stage (Figure 5C) using quantitative real time-PCR under NLD conditions. Most of the *Ghd10* mRNA accumulated at high levels in developing leaves (DL1) within the leaf sheath. The expression level of *Ghd10* was higher in DL1 than in older leaves (DL2, DL3 and DL4), but there was no significant difference in expression levels between DL3 and DL4 (Figure 5D). These results indicated that during leaf development, the expression of *Ghd10* in leaves gradually declined and was then maintained at a stable level. We also examined the expression patterns of *Ghd10* in WYJ7 roots, culms, leaf blades, panicles, spikelets and rachises at the heading stage by RT-PCR. *Ghd10* was expressed in all of the tissues examined, with relatively high levels of expression in the spikelets, panicles and rachises and lower expression levels in the culms and mature leaf blades, but the transcripts were difficult to detect in the roots (Figure 5E). The observation that *Ghd10* expression varied in an organ-specific manner was consistent with the varied phenotypes observed in *ghd10*. These results suggest that *Ghd10* first accumulates in developing leaves and interacts with an endogenous or exogenous signal to control the induction of the transition from vegetative to reproductive development in the early vegetative growth stage, and this gene is highly expressed in panicles to regulate panicle development after the transition to the reproductive growth stage occurs.

### *Ghd10* regulates the expression levels of floral pathway genes

**Figure 6:**
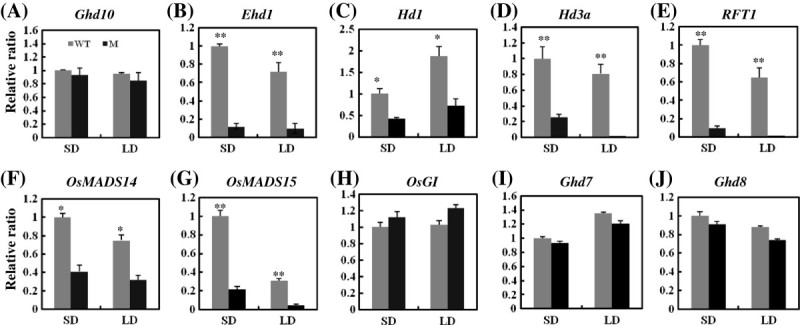
**Expression analysis of floral pathway genes in** ***ghd10*** **. (A**–**J)** The expression levels of *Ghd10* **(A)**, *Ehd1* **(B)**, *Hd1* **(C)**, *Hd3a* **(D)**, *RFT1* **(E)**, *OsMADS14* **(F)**, *OsMADS15* **(G)**, *OsGI* **(H)**, *Ghd7* **(I)** and *Ghd8* **(J)** in the *ghd10* mutant and wild-type plants under SD and LD conditions. Leaves were harvested 0.5 hour after dawn from 10-week-old plants under both SD and LD conditions. The experiment was repeated three times. The relative mRNA level of each gene was normalized to the level of rice ubiquitin mRNA. Vertical bars indicate the standard deviation of three measurements. * and ** represented the significant difference (P < 0.05) and extremely significant difference (P < 0.01), respectively.

To examine the accumulation of mRNAs of *Ghd10*, *Ehd1*, *Hd1*, *Hd3a, RFT1* and other flowering-related genes during development, we harvested developed leaves from wild-type and *ghd10* plants at the heading stage and analyzed the leaves by quantitative RT-PCR. The samples were collected 0.5 h after dawn under SD and LD conditions (Figure 6A). There was no significant difference in the levels of *Ghd10* mRNA accumulation between *ghd10* and wild-type plants under SD or LD conditions. These results suggest that the point mutation in *Ghd10* may not affect the transcription level of *Ghd10*, suggesting that the resulting change in a single amino acid residue in Ghd10 may affect the advanced structure and function of this protein. By contrast, the expression levels of *Ehd1* and *Hd1* dramatically decreased under both SD and LD conditions. A previous study demonstrated that the combination of mutant alleles of *Hd1* and *Ehd1* in T65 rice can reduce the number of primary branches in the panicle, resulting in smaller spikelet numbers per panicle (Endo-Higashi and Izawa 2011), which suggested that the combination of *hd1* and *ehd1* alleles, or the downregulated expression of *Hd1* and *Ehd1*, may increase the number of primary branches and the number of spikelets per panicle. To help confirm this hypothesis, we examined changes in the expression levels of *Hd1* and *Ehd1* in *ghd10* versus wild-type plants (Figure 6B, C). And the result of expression pattern of *Hd1* and *Ehd1* supported our speculation and previous reports (Endo-Higashi and Izawa 2011). *Hd3a*, *RFT1*, *OsMADS14* and *OsMADS15* encode key regulators in the floral pathway and function downstream of *Hd1* and *Ehd1*. The expression patterns of these genes matched that of *Hd1* and *Ehd1* (Figure 6D–G). The expression of *OsGI* slightly increased under both SD and LD conditions in *ghd10*, but the level of increase was not significant (Figure 6H). The expression levels of *Ghd7* and *Ghd8* were not significantly difference between WYJ7 and *gdh10* under both growth conditions (Figure 6I, J). While the expression levels of panicle development-related genes, such as *DEP1*, *FZP*, *LAX1* and *SP1*, were not significantly different between WYJ7 and *gdh10* under both growth conditions (Additional file 1: Figure S1). These results suggest that Ghd10 regulates flowering time, panicle development and yield formation may independent on Ghd7 and Ghd8.

## Discussion

### *Ghd10* controls yield-related traits in addition to flowering time

Recent studies have shown that flowering-related genes in rice, *Arabidopsis* and other plants play important roles in various physiological processes other than flowering. In rice, increased tillering occurs in transgenic rice plants expressing the gene encoding florigen Hd3a-GFP fusion protein under the control of phloem-specific promoters, in addition to accelerated flowering (Tamaki et al. 2007). Nano scale proteomics revealed the presence of regulatory proteins including three FT-like proteins, OsFTL12*,* OsRCN3 and OsMFT1, in rice phloem sap (Aki et al. 2008). Moreover, growth analysis of lateral shoots in mutants of *FLOWERING LOCUS T (FT)* and *TWIN SISTER OF FT (TSF)* in *Arabidopsis* reveals a delay in the onset of outgrowth and a reduction of the growth rate in *ft* plants under LD conditions and in *tsf* plants under SD conditions. In addition, two florigen genes, *FT* and *TSF*, play important roles in linking the floral transition and lateral shoot development to maximize the reproductive success of *Arabidopsis* plants. The promotion of lateral shoot development by these two florigen genes is independent of their effect on the floral transition of the primary shoot (Hiraoka et al. 2013). All of these studies suggest that flowering time and other traits can be affected by a signal gene in plants. The interaction between the two florigen genes may also influence yield-related traits in rice. As previously reported, *Hd1* promotes flowering under SD conditions while inhibiting flowering under LD conditions (Yano, et al. 2000). *Ehd1* promotes the floral transition preferentially under SD conditions, even in the absence of functional alleles of *Hd1* (Doi et al. 2004). In a study by Endo-Higashi and Izawa, four rice lines with different flowering-time genotypes (*hd1 ehd1*, *hd1 Ehd1*, *Hd1 ehd1* and *Hd1 Ehd1*) were grown under distinct photoperiod conditions. The authors compared the effects of flowering-time genes on traits related to plant architecture using genotype-treatment combinations that resulted in similar flowering times in the lines that were examined. The results showed that the combination of *Hd1* and *Ehd1* reduces the primary branch number in a panicle, resulting in a reduced spikelet number per panicle (Endo-Higashi and Izawa 2011), which demonstrates that the interaction between *Hd1* and *Ehd1* influences primary branch development independent of controlling flowering time. In the current study, the *ghd10* plants displayed outstanding yield traits, including increased panicle length, primary branch number and plant height, in addition to late heading date under SD conditions. The expression levels of two florigen genes (*Hd3a* and *RFT1*)*,* two rice *AP1* genes (*OsMADS14* and *OsMADS15*) and the combination of *Ehd1* and *Hd1* dramatically declined in *ghd10* plants under both SD and LD conditions. Combining the results of expression analysis and phenotypic variation analysis in *ghd10* plants, we conclude that the *ghd10* allele increases primary branch number, panicle length and plant height, and this allele also controls flowering time in response to SD growth conditions.

### The long vegetative growth period increases yield formation potential

Flowering time, one of the most important agronomic traits in rice, not only determines or influences the growth period of rice, but it also affects other agronomic traits (Hori et al. 2012). In studies of crop productivity, flowering time control was often associated with yield-related traits, as a prolonged vegetative period results in greater biomass accumulation, which can be conductive to the accumulation of more photosynthetic products. In fact, *Ghd7* has been shown to affect grain number in field tests (Xue et al. 2008). *Ghd7*, which was isolated from natural rice variants, encodes a CCT-domain protein and has major effects on heading date, plant height and the number of grains per panicle. Under LD conditions, the expression of *Ghd7* is enhanced, leading to delayed flowering time and increased plant height and panicle size. In a study comparing 19 genotypes of rice from rice growing areas representing a wide range of geographic regions in Asia, rice varieties with early heading, reduced plant height and panicle size were shown to have a deletion of the *Ghd7* locus. When *Ghd7* was transferred to plants, the transformants exhibited delayed flowering, increased plant height and panicle size (Xue et al. 2008).

Rice *Ghd8/DTH8*, a major QTL with pleiotropic effects on grain yield, heading date and plant height, encodes the OsHAP3 subunit of a CCAAT-box binding protein (HAP complex). *Ghd8/DTH8* is sensitive to photoperiod, and a mutation in this gene makes a plant insensitive to photoperiod and reduces plant height. Under LD conditions, plants with a deletion of *Ghd8/DTH8* exhibit delayed heading date and an increased plant height and number of grains per panicle when a functional *Ghd8/DTH8* allele is transferred to the plant (Wei et al. 2010; Yan et al. 2011). Here, we showed the *Ghd10* mutation has pleiotropic effects on grain yield, heading date and plant height and functions like *Ghd7* and *Ghd8/DTH8*. A longer vegetative growth period allows more photosynthate (the source) to be transferred to grains, i.e., the sink capacity increases, after flowering. When a plant has a large sink capacity and the flow between the sink and the source is unimpeded, the yield formation potential increases.

### The allele *ghd10* improves panicle traits in rice

Previous studies have revealed that the functions of rice *Ehd2/RID1/OsID1* and maize *ID1* are roughly conserved with respect to their role in flowering promotion and the tissue-specific expression of these genes, as there is high identity (82%) between their zinc finger domains (Matsubara et al. 2008; Park et al. 2008; Wu et al. 2008; Colasanti et al. 1998; Wong and Colasanti 2007). However, there are some functional differences between these genes. For instance, severe *id1* mutants in maize exhibit an abnormal transition to floral development and produce aberrant inflorescences with vegetative shoot characteristics (Colasanti et al. 1998). Such a morphological aberration has not been observed in *ehd2, rid1* or *Osid1* plants (Matsubara et al. 2008; Park et al. 2008; Wu et al. 2008). Therefore, species specificity of the genetic control of floral development may result in differences in the phenotypes caused by the mutation of orthologous gene, such as rice *Ehd2/RID1/OsID1* and maize *ID1*.

Phenotypic differences caused by the mutation of allelic genes also exist between subspecies or varieties of rice (Matsubara et al. 2008; Park et al. 2008; Wu et al. 2008). In this study, *Ghd10* was cloned using a map-based approach, and the locus was shown to encode a zinc finger transcript factor by transgenic complementation studies. Furthermore, the *Ghd10* locus of the *ghd10* mutant was shown to be allelic to *Ehd2/RID1/OsID1*. However, there were differences between the phenotypes of *ghd10* that we observed and the phenotypes reported for *ehd2, rid1* and *osid1*. In rice *ehd2* plants, a 4-bp insertion within the second exon of the putative zinc finger protein encoded by the *Ehd2* allele resulted in a premature stop codon in the open reading frame of this gene. The change in the coding sequence gave rise to plants with late flowering and smaller panicles compared with the wild type (Matsubara et al. 2008). In another study, *rid1* plants exhibited “eternal” vegetative growth and a never-flowering phenotype under both SD and LD conditions, which was caused by a T-DNA insertion in the second intron of the gene encoding a putative zinc finger protein (Wu et al. 2008). However, in the current study, the point mutation in the zinc finger motif of the Ehd2/RID1/OsID1 protein in the *ghd10* plants conferred obvious advantages to yield formation traits, including increased plant height, panicle length, primary branch number and number of spikelets per panicle, compared with the wild type and three other allelic mutants. This difference may have resulted from differences in the position/interval of the mutation in the coding sequences or differences in the genetic background of the allelic mutants examined; this topic remains to be explored in the future. We also plan to excavate as many *ghd10* alleles as possible and screen the more favorable alleles for rice production practice using a functional genomics approach.

Overall, the cloning of *Ghd10* has provided a rare opportunity for studying the molecular mechanisms underlying the association between yield-related traits and flowering. The *ghd10* allele is a useful resource for rice breeding to improve panicle traits in rice grown in tropical and low-latitude areas.

## Conclusion

The *ghd10* mutant is characterized by delayed flowering time, tall stalks, increased panicle length and primary branch number and other phenotypes. Map-based cloning revealed that *Ghd10* encodes a transcription factor with Cys-2/His-2-type zinc finger motifs orthologous to the *INDETERMINATE1 (ID1)* gene in maize. Expression analysis indicated that *Ghd10* acts as a flowering promoter and influences plant height and panicle development by regulating the expression levels of some flowering-related genes, such as *Ehd1*, *Hd1*, *OsMADS15* and others. The *ghd10* allele represents a useful resource for improving yield-related traits in rice bred for tropical and low-latitude areas.

## Materials and methods

### Plant materials and growth conditions

The *ghd10* mutant was derived from an M_2_ population of the *japonica* rice cultivar Wuyunjing 7 (WYJ7) after EMS mutagenesis (After soaking for 24 hours, transfer the seeds into 1.5% (v/v) EMS solution at 28 degree environmental temperature and treat with 12 hours, then washing the seeds 10 hours in flowing water and accelerating germinations. See the Ref. of Guo et al. (2006)). The *ghd10* mutant and progeny all exhibited delayed flowering time, increased primary branch number and tall stalks, inherited. The *japonica* cultivars Nipponbare (NIP), Wuyunjing 7 (WYJ7) and Chunjiang 06 (CJ06) and the *indica* cultivar Nanjing 06 (NJ06) were used for segregating population construction. All plants were grown in a paddy field at the China National Rice Research Institute (CNRRI), Fuyang, Zhejiang Province, China and Lingshui, Hainan Province, China.

### Photoperiod sensitivity test

To determine whether the mutation of *ghd10* was associated with photoperiod, 25-day-old *ghd10* and WYJ7 rice seedlings were cultivated in growth chambers (SANYO, Versatile Environmental Test Chamber, MLR-351H) under both continuous SD conditions (9.5 h light/14.5 h dark) and LD conditions (14.5 h light/ 9.5 h dark) with diurnal variation of temperature and relative humidity (Additional file 1: Table S1). The days to heading of *ghd10* and WYJ7 under both growth conditions were recorded.

### Genetic analysis and map-based cloning

For genetic analysis to determine whether a dominant or recessive, single or multiple gene controls the *ghd10* phenotype, reciprocal crosses between *ghd10* and the *japonica* cultivars NIP, WYJ7 and CJ06 were conducted. The F_2_ segregation populations were used for a χ^2^ test.

For map-based cloning, an F_2_ segregation population for mapping derived from a cross between the *ghd10* mutant and the *indica* cultivar NJ06 was constructed to identify the gene in the mutants. The parents and 6,846 F_2_ individuals were planted in a paddy field, among which 978 with the mutant phenotype were used to map the *Ghd10*. PCR genotyping was carried out using a DNA bulk-pool from 36 *ghd10*/NJ06 F_2_ individuals with the mutant phenotype, and a total of 136 SSR and STS markers scattered among all of the rice chromosomes were used to determine the approximate map position of the *ghd10* locus on the rice chromosomes according to Temnykh et al. (2000) and McCouch et al. (2002).

### DNA extraction and molecular marker analysis

Total genomic DNA was extracted from fresh leaves using the cetyltrimethylammonium bromide (CTAB) method with minor modifications. For mapping, SSR markers were obtained from the Gramene database (http://www.gramene.org). PCR-based STS and InDel markers were developed based on the sequence differences between *japonica* rice variety Nipponbare and *indica* variety 9311 (http://www.gramene.org/resources/). The primer sequences of the molecular markers used are listed in Additional file 1: Table S3. Primers flanking the InDel and SNP polymorphisms were designed using the Primer Premier 5.0 program and tested on the parent varieties by agarose gel electrophoresis.

### Complementation test

A 6,334-bp genomic DNA fragment containing the entire *Ghd10* coding region and upstream and downstream sequences of *Ghd10* was subcloned from the BAC clone AC027658 and inserted into the binary vector pCAMBIA1300 to generate the transformation vector pGhd10 for use in the complementation test. The pGhd10 and pCAMBIA1300 (pCK) plasmids were introduced into *ghd10* by *Agrobacterium*-mediated transformation using *Agrobacterium tumefaciens* strain EHA105.

### Subcellular localization of Ghd10

To investigate the subcellular localization of Ghd10, the *Ghd10* ORF without a termination codon was cloned into the pCaMV35S-GFP binary vector, placing Ghd10 upstream of the GFP coding sequence to create an in-frame fusion of *Ghd10* cDNA and the GFP reporter gene. The fusion constructs, as well as the control, were transformed into tobacco (*Nicotiana benthamiana*) epidermal leaf cells by *Agrobacterium*-mediated injection. The transformed tobacco epidermal cells were incubated for approximately 48 h at 24°C under 10 h dark/14 h light conditions. The cells were then examined under a confocal fluorescence microscope (Carl Zeiss, LSM 780).

### RNA extraction and quantitative real-time PCR analysis

Total RNA was extracted from the leaves of plants that were grown under SD or LD conditions using a Total RNA Extraction Kit (Axygen, cat No,AP-MN-MS-RNA-250). Total RNA was treated with RNase-free DNase (Promega; http://www.promega.com) and used for complementary DNA synthesis using a ReverTra Ace qPCR-RT Kit (TOYOBA, Japan) as described by the manufacturer. Real-time PCR was performed using 2 × SYBR Green PCR Master Mix (Applied Biosystems) in an Applied Biosystems 7900HT Real-Time PCR System with at least three PCR replicates per sample. The PCR conditions were 2 min at 50°C, then 10 min at 95°C, followed by 40 cycles of 15 s at 95°C and 1 min at 60°C. The relative expression level of each transcript was compared with that of *RUBQ2*. The specific primers used for quantification of *Hd3a, RFT1, OsMADS15, OsMADS14, Hd1, Ehd1, Ghd7, Ghd8, DEP1, FZP, LAX1, SP1* and *RUBQ2* mRNA expression are listed in Additional file 1: Table S4.

To analyze the expression pattern of *Ghd10*, total RNA was extracted from roots, culms, leaves, leaf sheaths, panicles, rachises and spikelets of plants at the heading stage and from leaves at various developmental stages from plants at the tillering stage using a Total RNA Extraction Kit (Axygen, cat No,AP-MN-MS-RNA-250). The specific primers 5′-CGACAATAGCTCGATCGCC-3′ and 5′-AAGCCCGAAGCTGACACTGT-3′ were used to quantify the expression of *Ghd10* mRNA. The relative expression levels in different tissues were determined to compare the expression of *Ghd10* with that of *RUBQ2*.

## Electronic supplementary material

Additional file 1: Table S1: The diurnal variation of light and temperature in the illumination incubator. **Table S2:** Genetic analysis of *ghd10.* **Table S3:** Molecular markers used for *Ghd10* mapping and sequencing. **Table S4:** Real-time PCR primers used in this study. **Figure S1:** Transcriptional levels in *ghd10* and WYJ7 of (A) *DEP1*, (B) *FZP*, (C) *LAX1*, (D) *SP1*. Data are displayed as the ratio of expression to rice *RUBQ2* RNA, data given as mean ± standard error. All assays were repeated at least three times. (PPT 416 KB)

Below are the links to the authors’ original submitted files for images.

Authors’ original file for figure 1

Authors’ original file for figure 2

Authors’ original file for figure 3

Authors’ original file for figure 4

Authors’ original file for figure 5

Authors’ original file for figure 6
