# Supplementary material for: A point mutation in the zinc finger motif of RID1/EHD2/OsID1 protein leads to outstanding yield-related traits in japonica rice variety Wuyunjing 7
Source: Rice (N Y). 2013 Oct 18;6:24. doi: 10.1186/1939-8433-6-24 (PMC4883695; doi:10.1186/1939-8433-6-24)
Supplement: Supplementary file 1 — Additional file 1: Table S1: The diurnal variation of light and temperature in the illumination incubator. Table S2: Genetic analysis of ghd10. Table S3: Molecular markers used for Ghd10 mapping and sequencing. Table S4: Real-time PCR primers used in this study. Figure S1: Transcriptional levels in ghd10 and WYJ7 of (A) DEP1, (B) FZP, (C) LAX1, (D) SP1. Data are displayed as the ratio of expression to rice RUBQ2 RNA, data given as mean ± standard error. All assays were repeated at least three times. (PPT 416 KB) [file 12284_2013_60_MOESM1_ESM.ppt]

## Slide 1
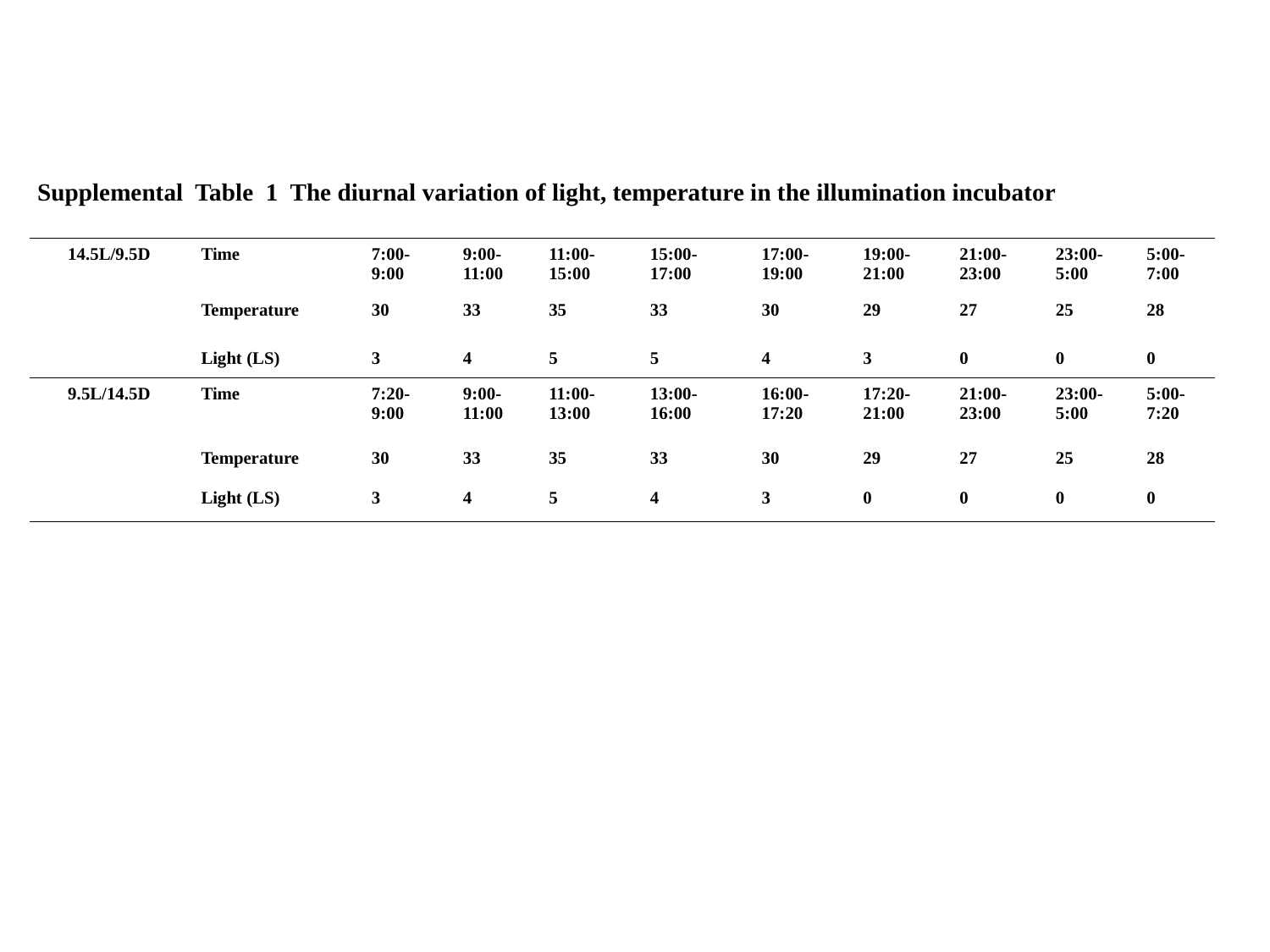

Supplemental Table 1 The diurnal variation of light, temperature in the illumination incubator
| 14.5L/9.5D | Time | 7:00-9:00 | 9:00-11:00 | 11:00-15:00 | 15:00-17:00 | 17:00-19:00 | 19:00-21:00 | 21:00-23:00 | 23:00-5:00 | 5:00-7:00 |
| --- | --- | --- | --- | --- | --- | --- | --- | --- | --- | --- |
| | Temperature | 30 | 33 | 35 | 33 | 30 | 29 | 27 | 25 | 28 |
| | Light (LS) | 3 | 4 | 5 | 5 | 4 | 3 | 0 | 0 | 0 |
| 9.5L/14.5D | Time | 7:20-9:00 | 9:00-11:00 | 11:00-13:00 | 13:00-16:00 | 16:00-17:20 | 17:20-21:00 | 21:00-23:00 | 23:00-5:00 | 5:00-7:20 |
| | Temperature | 30 | 33 | 35 | 33 | 30 | 29 | 27 | 25 | 28 |
| | Light (LS) | 3 | 4 | 5 | 4 | 3 | 0 | 0 | 0 | 0 |

## Slide 2
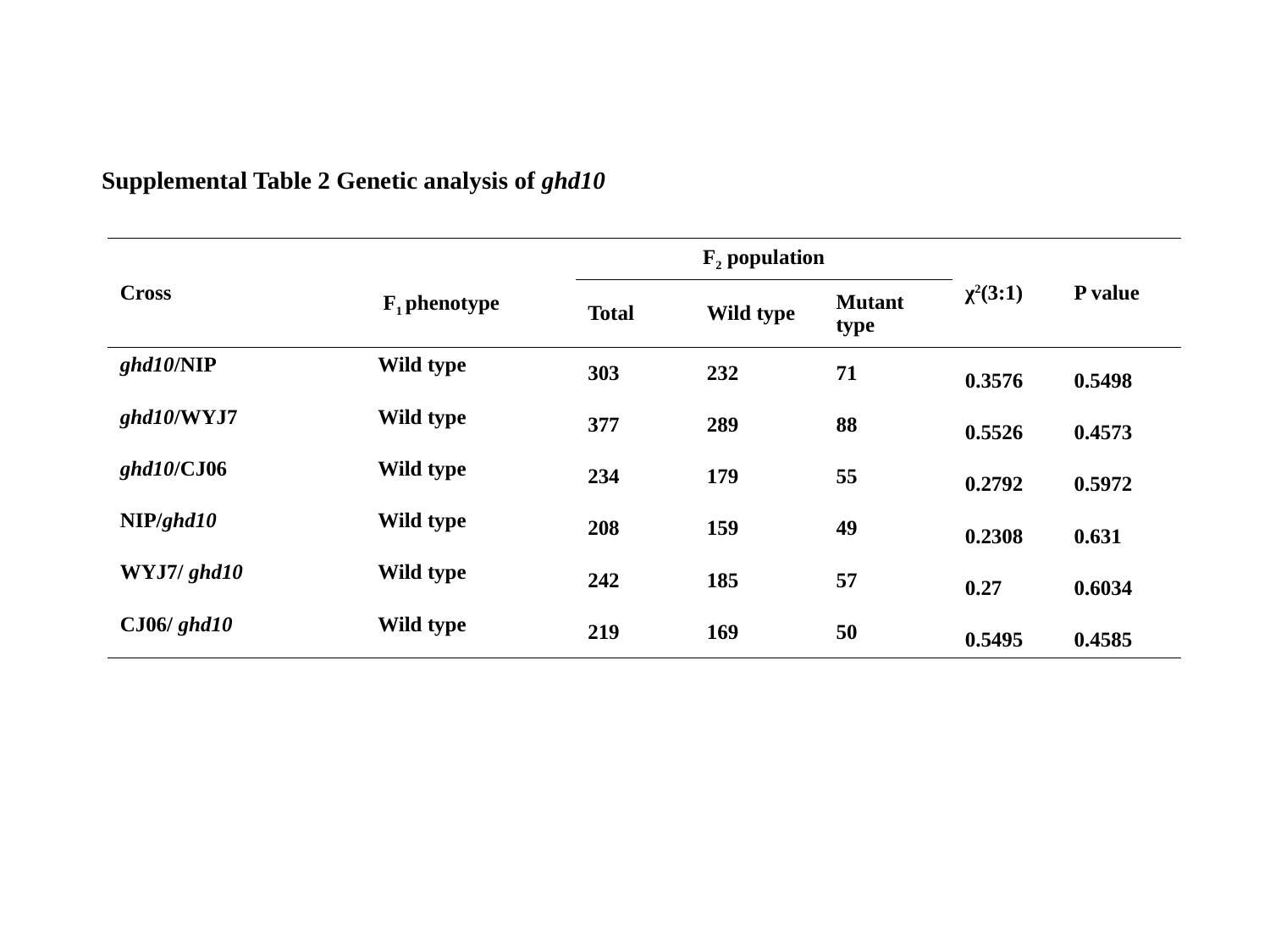

Supplemental Table 2 Genetic analysis of ghd10
| Cross | F1 phenotype | F2 population | | | χ2(3:1) | P value |
| --- | --- | --- | --- | --- | --- | --- |
| | | Total | Wild type | Mutant type | | |
| ghd10/NIP | Wild type | 303 | 232 | 71 | 0.3576 | 0.5498 |
| ghd10/WYJ7 | Wild type | 377 | 289 | 88 | 0.5526 | 0.4573 |
| ghd10/CJ06 | Wild type | 234 | 179 | 55 | 0.2792 | 0.5972 |
| NIP/ghd10 | Wild type | 208 | 159 | 49 | 0.2308 | 0.631 |
| WYJ7/ ghd10 | Wild type | 242 | 185 | 57 | 0.27 | 0.6034 |
| CJ06/ ghd10 | Wild type | 219 | 169 | 50 | 0.5495 | 0.4585 |

## Slide 3
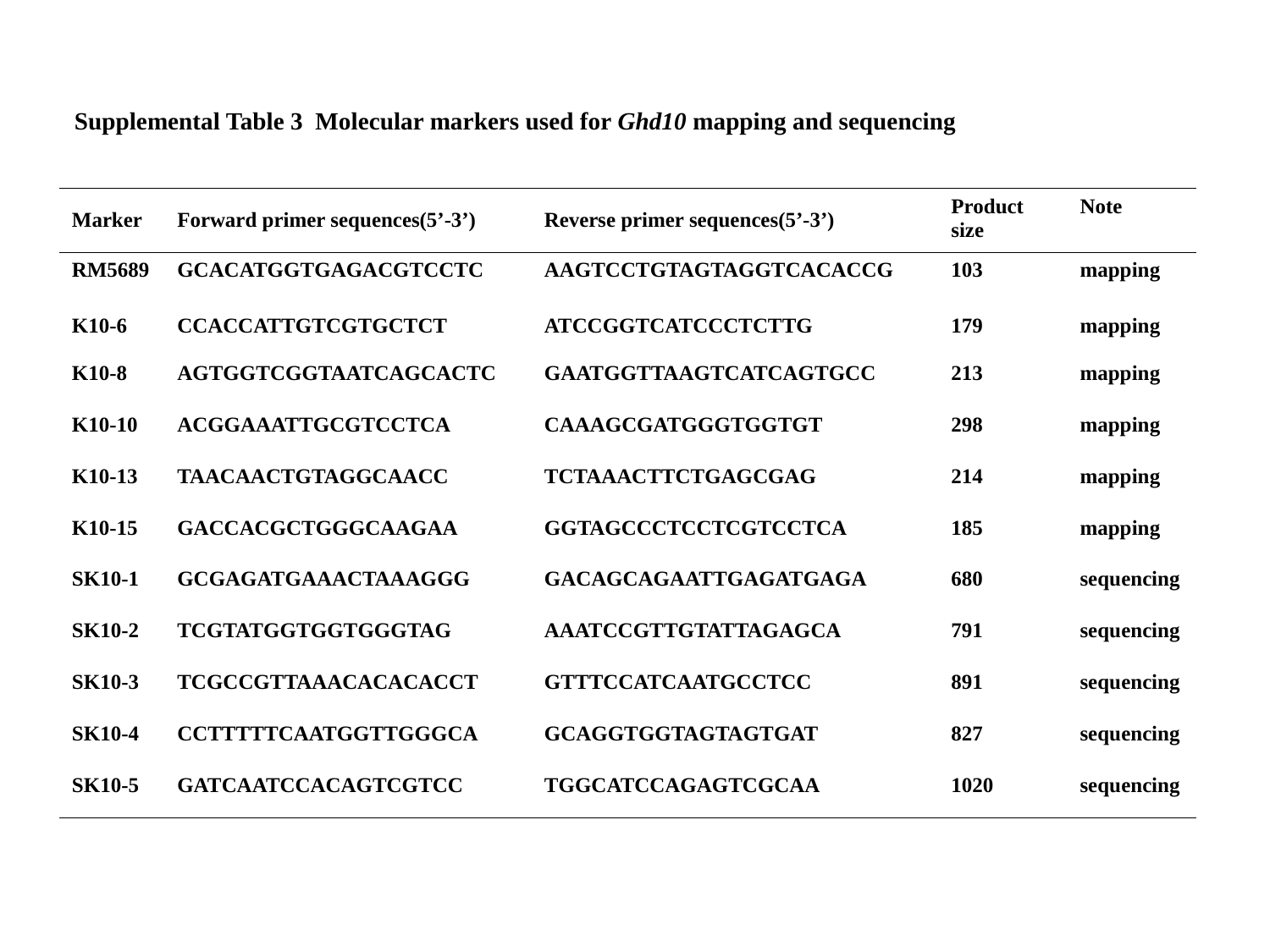

Supplemental Table 3 Molecular markers used for Ghd10 mapping and sequencing
| Marker | Forward primer sequences(5’-3’) | Reverse primer sequences(5’-3’) | Product size | Note |
| --- | --- | --- | --- | --- |
| RM5689 | GCACATGGTGAGACGTCCTC | AAGTCCTGTAGTAGGTCACACCG | 103 | mapping |
| K10-6 | CCACCATTGTCGTGCTCT | ATCCGGTCATCCCTCTTG | 179 | mapping |
| K10-8 | AGTGGTCGGTAATCAGCACTC | GAATGGTTAAGTCATCAGTGCC | 213 | mapping |
| K10-10 | ACGGAAATTGCGTCCTCA | CAAAGCGATGGGTGGTGT | 298 | mapping |
| K10-13 | TAACAACTGTAGGCAACC | TCTAAACTTCTGAGCGAG | 214 | mapping |
| K10-15 | GACCACGCTGGGCAAGAA | GGTAGCCCTCCTCGTCCTCA | 185 | mapping |
| SK10-1 | GCGAGATGAAACTAAAGGG | GACAGCAGAATTGAGATGAGA | 680 | sequencing |
| SK10-2 | TCGTATGGTGGTGGGTAG | AAATCCGTTGTATTAGAGCA | 791 | sequencing |
| SK10-3 | TCGCCGTTAAACACACACCT | GTTTCCATCAATGCCTCC | 891 | sequencing |
| SK10-4 | CCTTTTTCAATGGTTGGGCA | GCAGGTGGTAGTAGTGAT | 827 | sequencing |
| SK10-5 | GATCAATCCACAGTCGTCC | TGGCATCCAGAGTCGCAA | 1020 | sequencing |

## Slide 4
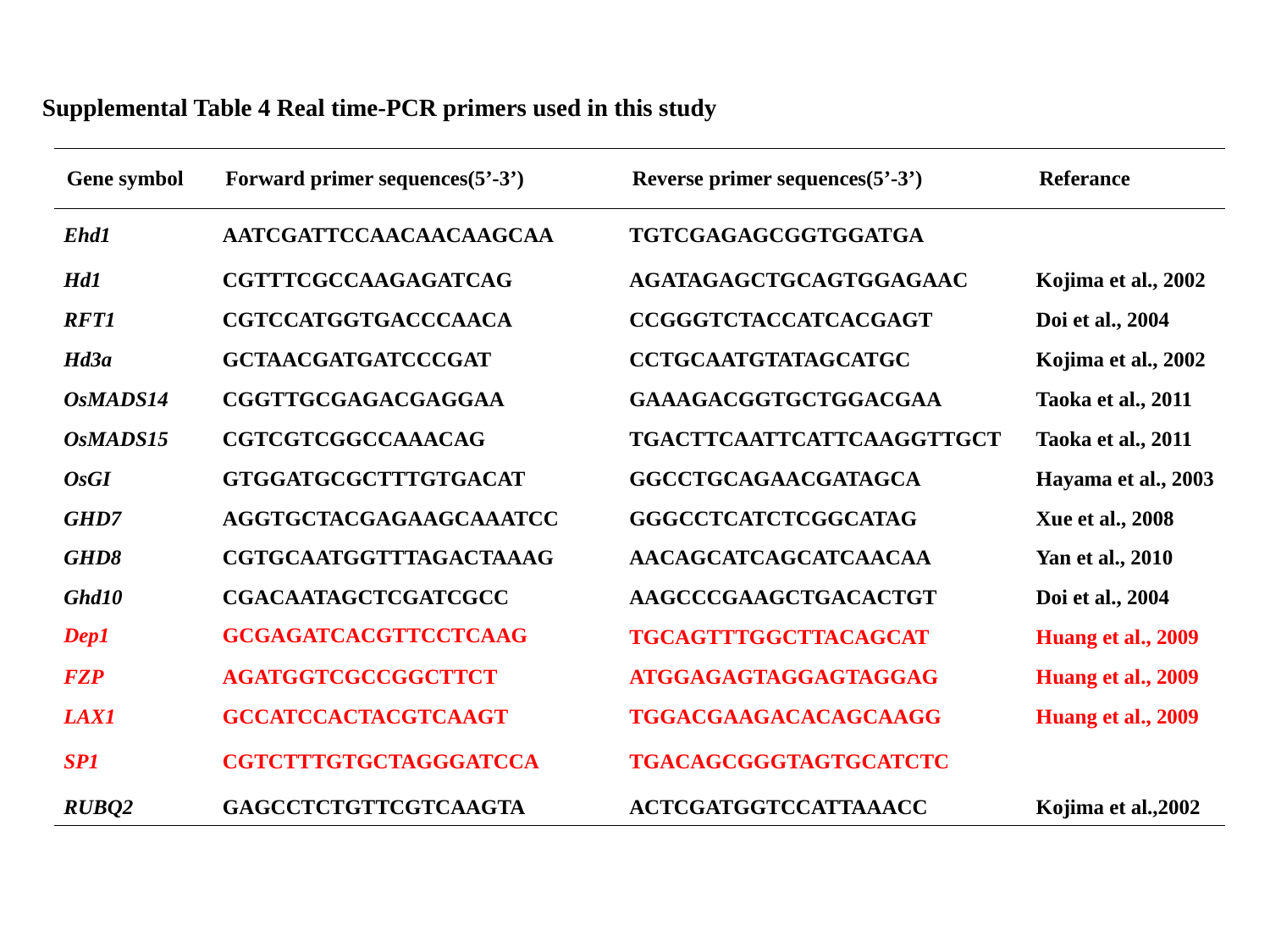

Supplemental Table 4 Real time-PCR primers used in this study
| Gene symbol | Forward primer sequences(5’-3’) | Reverse primer sequences(5’-3’) | Referance |
| --- | --- | --- | --- |
| Ehd1 | AATCGATTCCAACAACAAGCAA | TGTCGAGAGCGGTGGATGA | |
| Hd1 | CGTTTCGCCAAGAGATCAG | AGATAGAGCTGCAGTGGAGAAC | Kojima et al., 2002 |
| RFT1 | CGTCCATGGTGACCCAACA | CCGGGTCTACCATCACGAGT | Doi et al., 2004 |
| Hd3a | GCTAACGATGATCCCGAT | CCTGCAATGTATAGCATGC | Kojima et al., 2002 |
| OsMADS14 | CGGTTGCGAGACGAGGAA | GAAAGACGGTGCTGGACGAA | Taoka et al., 2011 |
| OsMADS15 | CGTCGTCGGCCAAACAG | TGACTTCAATTCATTCAAGGTTGCT | Taoka et al., 2011 |
| OsGI | GTGGATGCGCTTTGTGACAT | GGCCTGCAGAACGATAGCA | Hayama et al., 2003 |
| GHD7 | AGGTGCTACGAGAAGCAAATCC | GGGCCTCATCTCGGCATAG | Xue et al., 2008 |
| GHD8 | CGTGCAATGGTTTAGACTAAAG | AACAGCATCAGCATCAACAA | Yan et al., 2010 |
| Ghd10 | CGACAATAGCTCGATCGCC | AAGCCCGAAGCTGACACTGT | Doi et al., 2004 |
| Dep1 | GCGAGATCACGTTCCTCAAG | TGCAGTTTGGCTTACAGCAT | Huang et al., 2009 |
| FZP | AGATGGTCGCCGGCTTCT | ATGGAGAGTAGGAGTAGGAG | Huang et al., 2009 |
| LAX1 | GCCATCCACTACGTCAAGT | TGGACGAAGACACAGCAAGG | Huang et al., 2009 |
| SP1 | CGTCTTTGTGCTAGGGATCCA | TGACAGCGGGTAGTGCATCTC | |
| RUBQ2 | GAGCCTCTGTTCGTCAAGTA | ACTCGATGGTCCATTAAACC | Kojima et al.,2002 |

## Slide 5
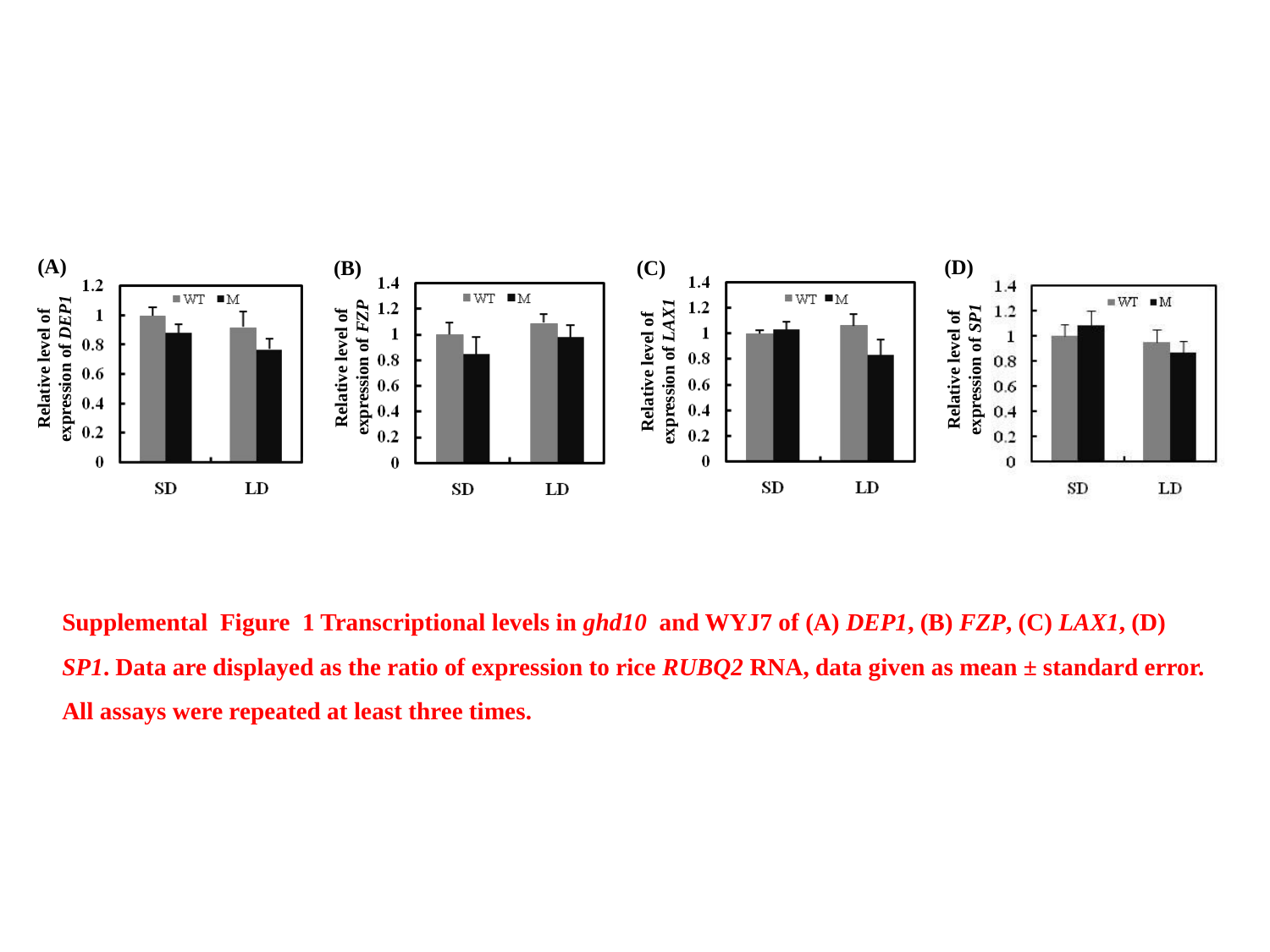

(A)
(D)
(B)
(C)
Relative level of expression of LAX1
Relative level of expression of FZP
Relative level of expression of DEP1
Relative level of expression of SP1
Supplemental Figure 1 Transcriptional levels in ghd10 and WYJ7 of (A) DEP1, (B) FZP, (C) LAX1, (D) SP1. Data are displayed as the ratio of expression to rice RUBQ2 RNA, data given as mean ± standard error. All assays were repeated at least three times.
