# Supplementary figures and images for: A point mutation in the zinc finger motif of RID1/EHD2/OsID1 protein leads to outstanding yield-related traits in japonica rice variety Wuyunjing 7
Source: Rice (N Y). 2013 Oct 18;6:24. doi: 10.1186/1939-8433-6-24 (PMC4883695; doi:10.1186/1939-8433-6-24)

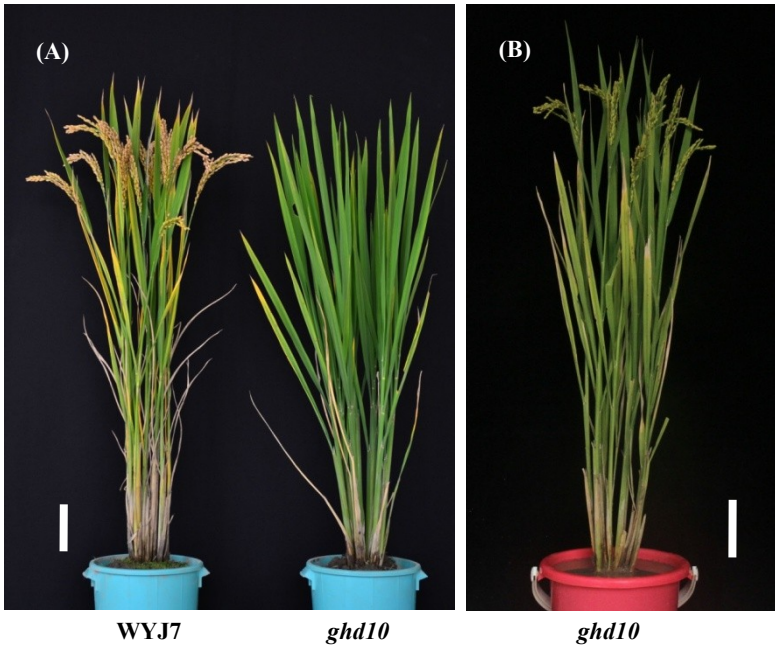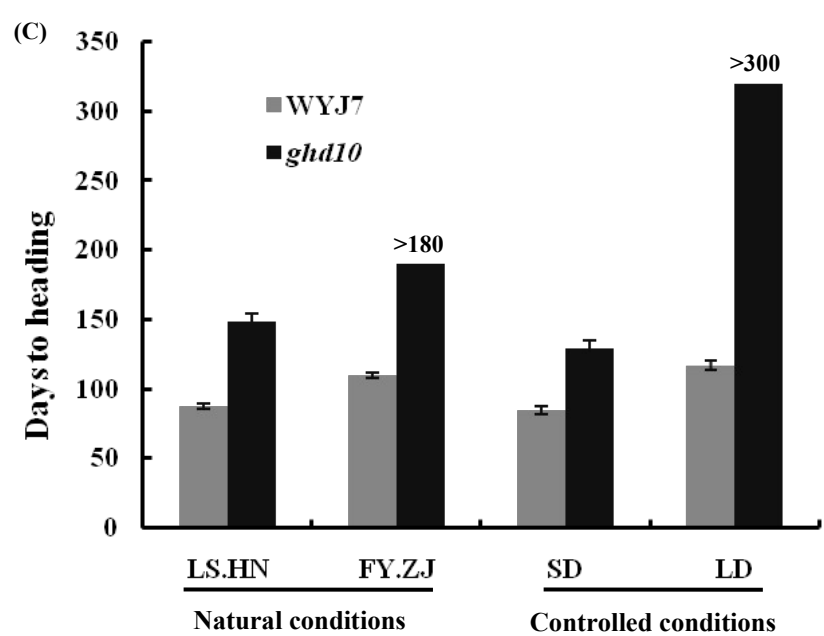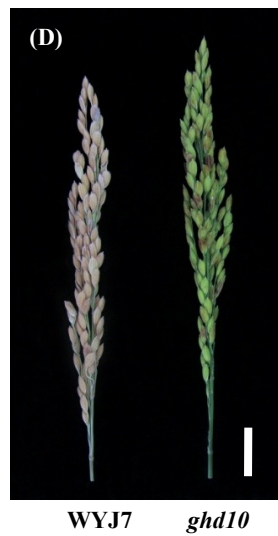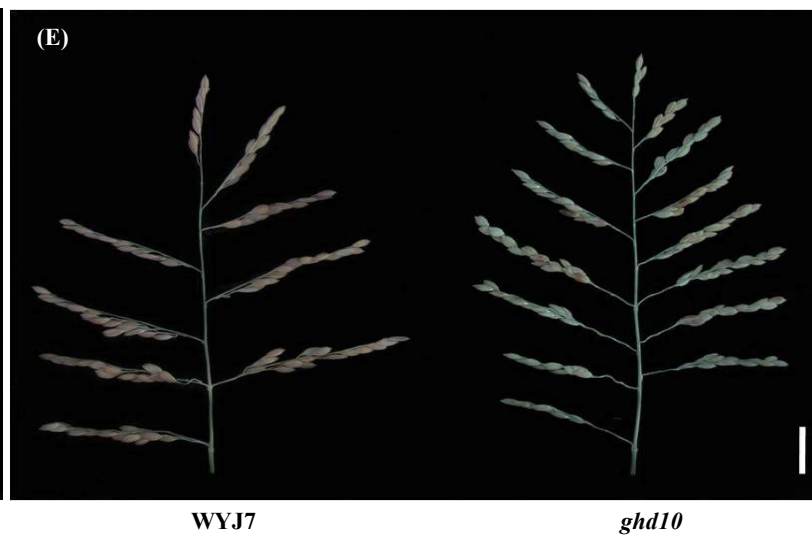

Supplement: Supplementary file 2 — Authors’ original file for figure 1 [file 12284_2013_60_MOESM2_ESM.pdf]

**(A)**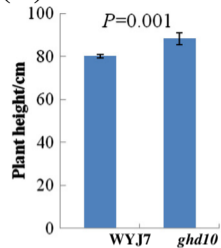**(B)**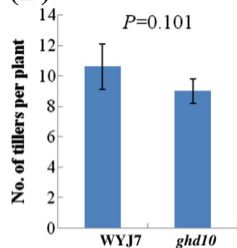**(C)**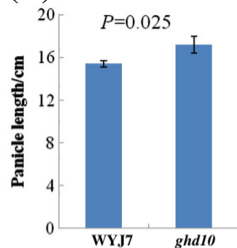**(D)**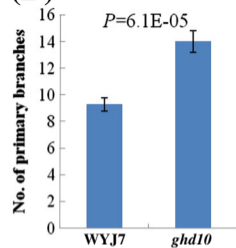**(E)**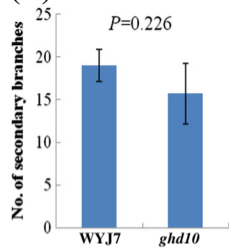**(F)**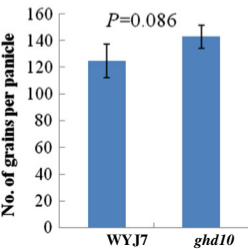**(G)**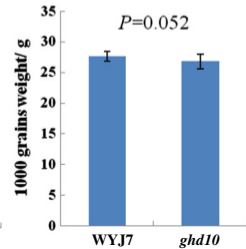**(H)**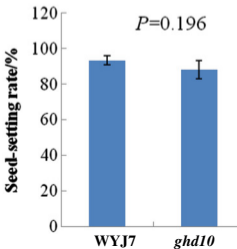**(I)**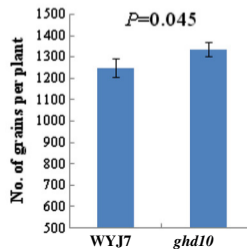**(J)**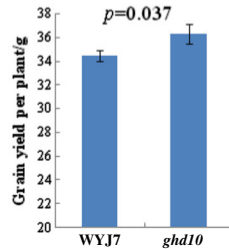

Supplement: Supplementary file 3 — Authors’ original file for figure 2 [file 12284_2013_60_MOESM3_ESM.pdf]

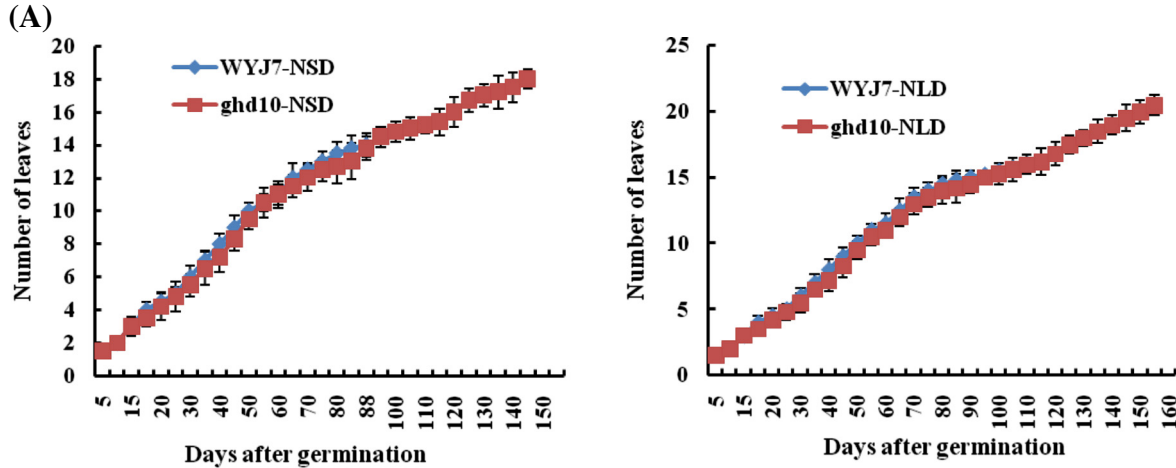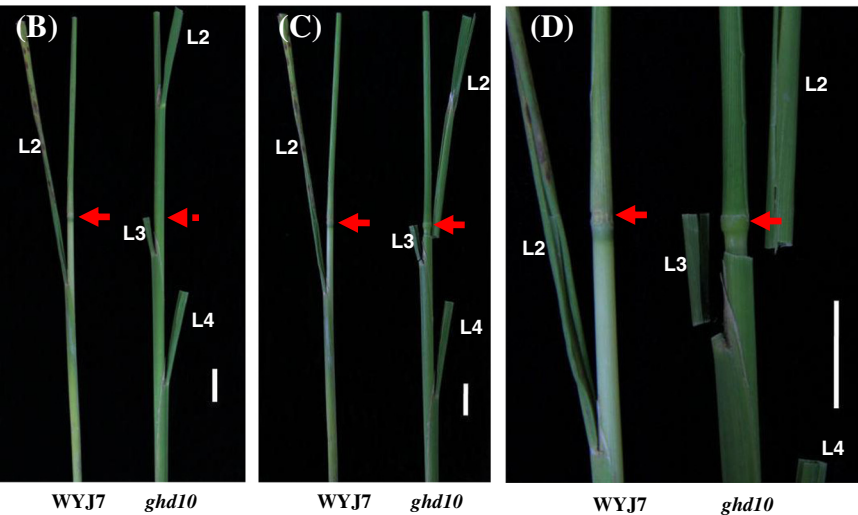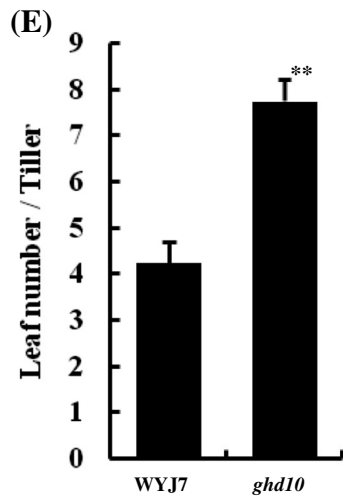

Supplement: Supplementary file 4 — Authors’ original file for figure 3 [file 12284_2013_60_MOESM4_ESM.pdf]

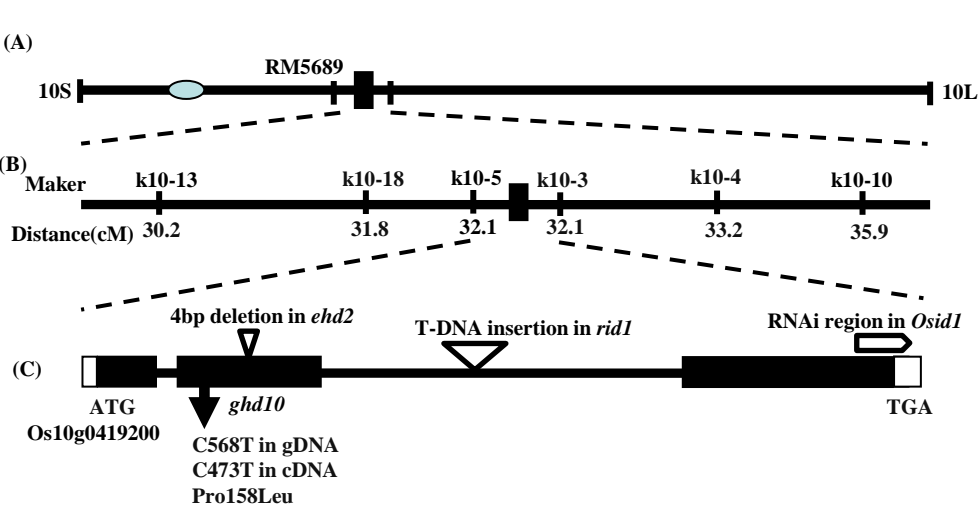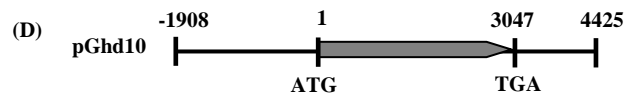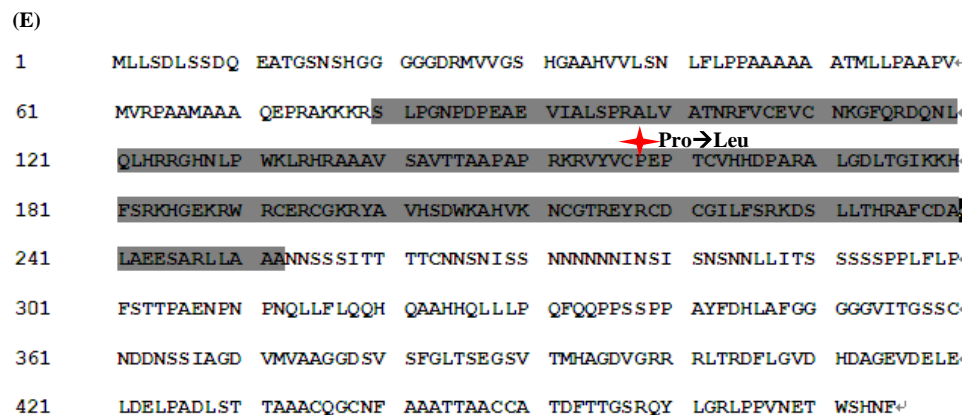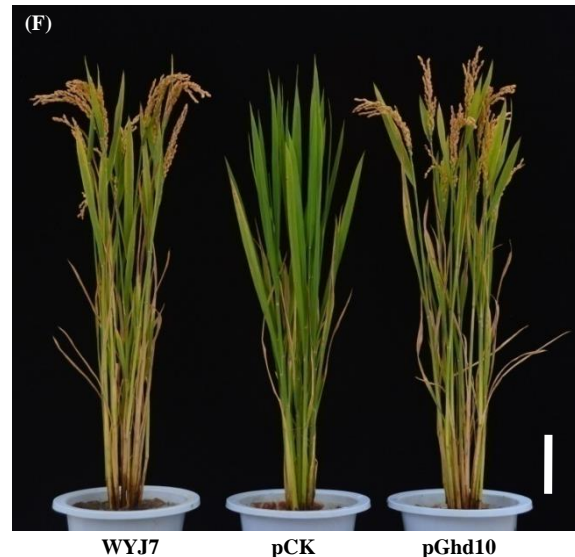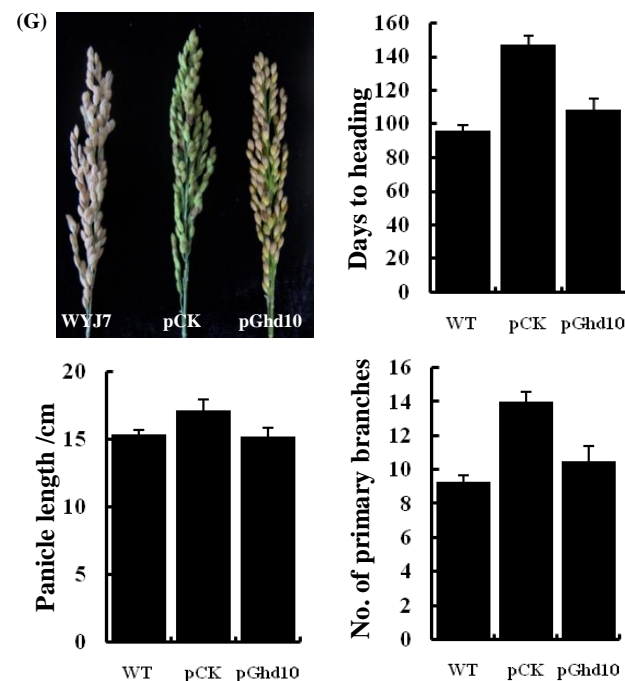

Supplement: Supplementary file 5 — Authors’ original file for figure 4 [file 12284_2013_60_MOESM5_ESM.pdf]

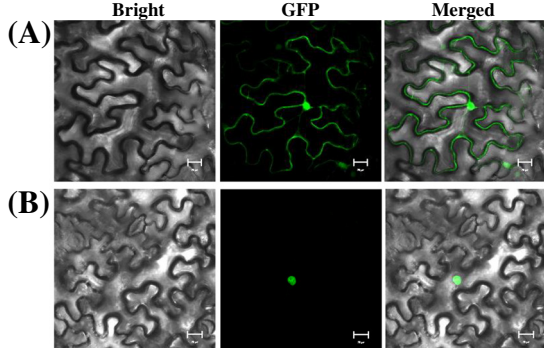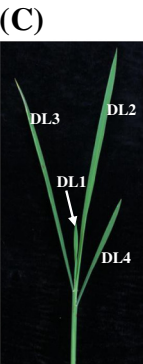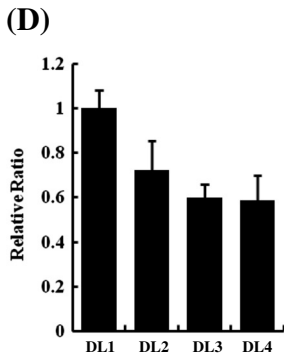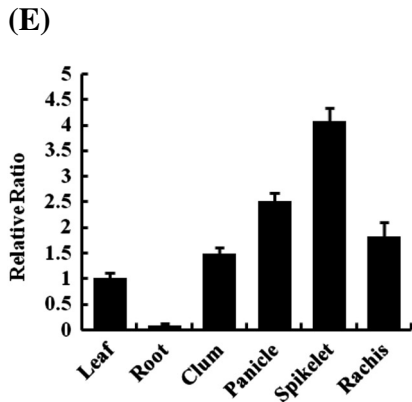

Supplement: Supplementary file 6 — Authors’ original file for figure 5 [file 12284_2013_60_MOESM6_ESM.pdf]

**(A)***Ghd10*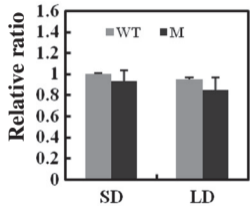**(B)***Ehd1*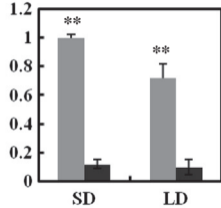**(C)***Hd1*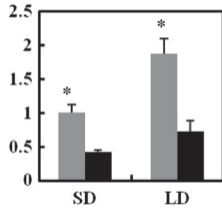**(D)***Hd3a*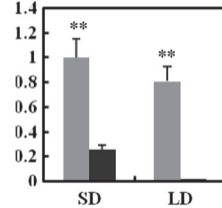**(E)***RFT1*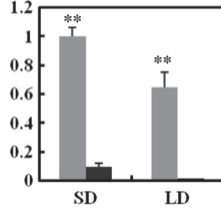**(F)***OsMADS14*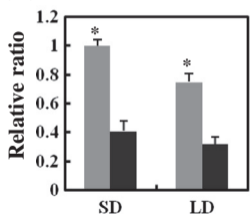**(G)***OsMADS15*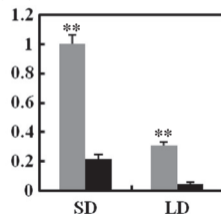**(H)***OsGI*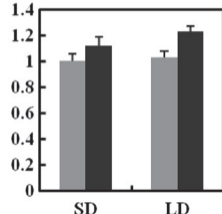**(I)***Ghd7*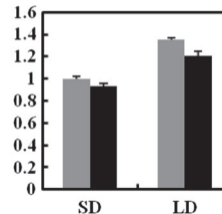**(J)***Ghd8*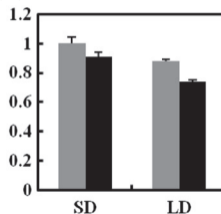

Supplement: Supplementary file 7 — Authors’ original file for figure 6 [file 12284_2013_60_MOESM7_ESM.pdf]
